# Effects of Enclosure Complexity and Design on Behaviour and Physiology in Captive Animals

**DOI:** 10.3390/ani14142028

**Published:** 2024-07-09

**Authors:** Andrew Smith, Paul Rose, Claudia Mettke-Hofmann

**Affiliations:** 1 School of Life Sciences, Faculty of Science and Engineering, Anglia Ruskin University, East Road, Cambridge CB1 1PT, UK; andrew.smith@aru.ac.uk; 2 Centre for Research in Animal Behaviour, Psychology, University of Exeter, Washington Singer, Perry Road, Exeter EX4 4QG, UK; p.rose@exeter.ac.uk; 3 WWT, Slimbridge Wetland Centre, Slimbridge, Gloucestershire GL2 7BT, UK; 4 School of Biological and Environmental Sciences, Liverpool John Moores University, James Parsons Building, Byrom Street, Liverpool L3 3AF, UK

## 1. Introduction

Individual animals in managed populations are subject to controlled social and physical environmental conditions that impact their behaviour patterns, choice of social associates, ability to experience positive welfare states, and ultimately their overall health status and quality of life. Previous research has shown the importance of ensuring that managed animals experience control and choice (i.e., have a sense of autonomy over what they can do and when) within the sphere of their housing, husbandry, and management regimes [1]. Likewise, inappropriate social groups that deviate from the structure of naturally occurring social environments at specific life stages can also induce stress and negatively impact enclosure usage [2,3]. Finally, impoverished captive environments that do not provide functional replication—i.e., recreation of the specific features of a species’ niche or provide resource access in an ecologically relevant way [4] or enable the performance of adaptive behaviour [5] can cause stress and reduce welfare. The managed environment provided for zoos, labs, and even companion species is more than the social conditions and physical features of the space directly experienced by the animals. The presence of humans, both familiar and unfamiliar, around an enclosure; the sights, sounds, and smells of the world outside of the animal’s housing; and the actual materials used for creating barriers or landscaping the areas used by animals all may influence the performance of specific behaviours.

Visitor presence has been largely documented as negatively influencing the behaviour and physiology of captive species [6,7,8], although other studies found no or positive effects [9,10]. In contrast, experiences of positive caregiver interactions in lab-housed species have improved welfare and behaviour [11]. Other factors, such as sounds from within and beyond an enclosure, can influence overall time activity patterns, enclosure usage, and stress physiology of captive species [12,13,14]. Likewise, other variables associated with, but not actually part of, the animal’s enclosure might promote or negate behavioural and physiological responses to social and physical environmental factors, which can ultimately cause species to thrive or fail under captive conditions. To unpick these complexities further, helping to advance animal care and management, this Special Issue was created to encourage dissemination of research into unexplored aspects across a range of holding conditions. It presents new research findings and their wider impacts across different aspects of animal management and poses new questions and important research extensions that still need to be investigated in order to fully comprehend how individuals’ physiological and behavioural responses are affected by both their directly and indirectly experienced environmental conditions.

## 2. Assembly of Articles

This Special Issue begins with de Azevedo et al.’s [15] cross-taxa introduction to the importance of complexity in enclosure design for captive animals. Their review outlines comprehensively how the many facets of environmental complexity, from basic structure (2D and 3D), vegetation, burrows, and shelters, to light levels, temperature, and humidity, coupled with social complexity in terms of con- and heterospecifics, and temporal variation, both diel and seasonal changes, need to be taken into account when designing or renovating enclosures. In the zoo context, enclosure design has shifted towards more natural enclosures that take species-specific requirements into account, but we also need to be aware of how differences between individuals, such as their age, sex, reproductive/biological state, or personality, influence their requirements and use of the captive environment and its microhabitats. These are explored through subsequent papers within this volume. Further, when constructing captive environments, the authors highlight that rather than basing them on our own perceptual capabilities, design should be considered from the perspective of the inhabiting species. The more complex and dynamic an environment, the greater array of choices it provides, allowing animals increased control over their immediate environment with ultimate benefits for their welfare and health. As Azevedo et al. [15] note, an individual’s choices or preferences may change as their social and physical environment varies over the course of the 24-hour period or with season, and understanding when, how, and which spaces animals use can provide greater insight into their needs and thus further inform future habitat design. Enclosure use evaluations, such as the spread of participation index (SPI) and post-occupancy evaluation (POE), are useful in this regard. This is particularly true when aiming to understand use differences between individuals or those related to changes to enclosures.

As zoos and other institutions holding captive animals move to more naturalistic and often larger enclosures, studies of what animals want, including at the level of the individual, are vital. The second [16] and third [17] papers use POE and SPI to relate habitat size and complexity to enclosure use and behaviour in king penguins (*Aptenodytes patagonicus*) and callimicos (*Callimico goeldii*), respectively. Fuller et al.’s [16] findings for penguins show larger, more complex enclosures can result in more welfare-positive (i.e., swimming) and less welfare-negative behaviour (i.e., agonistic interactions). Increased complexity can also provide animals the opportunity to express preferences (e.g., height or depth preferences) and behaviours not otherwise possible, such as laying recumbent in king penguins or terrestrial manipulative foraging in callimico, which require enclosures with ice and lose substrates, such as woodchip or soil, respectively. Bartlet, Grinsted, and Freeman’s [17] multi-institution study of enclosure use by callimico presents clear evidence of association of specific behaviours with different zones within enclosures and consistent findings for height use irrespective of the maximum height of the enclosure. As the authors note, this latter finding suggests that SPI should be used in conjunction with knowledge of the species’ natural habitat and zone use—allowing complexity to be targeted at zones the animals will use based on their natural propensities. They highlight the need for species-specific husbandry guidelines even for morphologically similar species. In addition, they suggest providing temporal complexity through changing some routes or pathways in the enclosure, such as branches or ropes in the case of arboreal species, to provide unpredictability as experienced in a naturally changing wild environment.

Then looking within species, Moise, Eccles, and Mettke-Hofmann [18] demonstrate differences between sexes and colour morphs in the preferences of Gouldian finches (*Chloebia gouldiae*). Their work highlights the importance of considering background in enclosure design, an understudied area. As Azevedo et al.’s [15] review paper note, the ability or otherwise to hide can have important welfare implications because background matching is a common camouflage strategy. Complexity, through the provision of varied backgrounds, allows individuals to choose their preferred background, which may differ depending on their sex and morph, as well as lighting conditions, time of day, season, and reproductive state. As the authors note, these choices may reflect properties of the animal’s natural habitat. The next paper by Mooney et al. [19] examines the implications when animals are not given choices. Their longitudinal study of zoo-housed Chilean flamingos (*Phoenicopterus chilensis*) showing the importance of free access to natural substrates in maintaining foot health again highlights the needs for enclosure design to take account of the physical properties of the animal’s in situ environment. Zoos are not the only captive environments, and the last paper by Lee et al. [20] follows on by looking at how housing environments can mitigate the stress consequences of an imposed change in lab-housed beagles. Their findings support the need for social and environmental complexity through enrichment in maintaining positive welfare.

## 3. Further Steps

The collection tackles some aspects of enclosure design, specifically the importance of substrate [16,17,19] but also the complexity of enclosures [15,16,18] and how it can be achieved through physical [17,20] or visual [18] enrichment. Future studies could focus on lighting. Little is known about how lighting within an enclosure can be used to create areas for different activities such as resting, nesting, foraging, or courtship (but see [21]). Furthermore, most species experience seasonal changes in light duration, which trigger major seasonal activities like reproduction, migration, or hibernation [22,23,24]. This could be an easy and powerful tool to synchronise partners yet is basically absent in laboratory animals with consistent lighting schedules and likely not commonly used in zoos. While there has been considerable research conducted on the effect of the light spectrum, specifically ultraviolet (UV) light, on courtship behaviour and mate choice [25,26], there is very little known about the importance of UV in a welfare context.

Another aspect that has not been covered here but is of interest is the environment outside the enclosure. It can either serve as visual enrichment (e.g., windows, wallpaper, or ornaments) or help camouflage what is behind (e.g., visitors in zoos). It could be another simple way of improving an animal’s environment, alleviating stress, and positively affecting behaviour; however, this field requires further research.

All studies in this Special Issue assessed welfare behaviorally. However, behavioural measures may not always accurately reflect underlying physiological stress responses [27]. With the increasing availability of non-invasive tools to measure physiological states, this is an area that needs more attention. Non-invasive measurements of stress hormones can be carried out by means of faeces [28], urine [29], or saliva [30] (but see [31]), although the latter requires contact with the animal. A relatively new method is thermal imaging for measuring changes in skin temperature, e.g., around the eye remotely and non-invasively, which correlates with physiological stress reactions (e.g., [32]). Combined with manipulations in the enclosure, these non-invasive tools to measure physiological state can help design less-stressful environments [33,34].

Last but not least, a larger range of taxa should be investigated across a range of holding conditions. The majority of relevant studies investigate enclosure design and use in zoological collections [35,36,37], with two studies included in this Special Issue also looking into laboratory settings [18,20]. Particularly, the latter requires more attention for non-rodent species; however, farm animals should be considered also. More attention should be paid to taxa, other than birds and mammals, such as reptiles, amphibia, and fish. These could also lead to the development of guidelines on how to structure environments for these groups in private enclosures.
